# Queen Alexandra's Court

**Published:** 1905-07-01

**Authors:** 


### QUEEN ALEXANDRA'S COURT.

In a pretty spot at Wimbledon surrounded by trees stands Queen Alexandra's Court, the name given to the homes lately built for officers' widows and daughters. The houses are built in three separate blocks, two of which face each other, the third facing the court and terrace. The exterior presents a pretty appearance, being built of red brick and white stone, with flowers flourishing from balconies on the second and third floors. The whole consists of 60 apartments or flats, 24 being already occupied.

A name indicator is fixed in the main entrance, and each flat has its own front door and is shut from the main entrances. The flats consist of one sitting-, two bedrooms, and a kitchen. The door, flooring and mantelpiece of the sitting-room are of oak. There are two windows, provided with green blinds. The lighting is by electricity. The prevailing colour throughout the flats is green. The kitchen is a fair size with a red tiled floor. For cooking purposes there is a gas-stove, also a small fireplace which would be convenient should the room be wanted for other purposes than that of a kitchen. The sink is in the kitchen, but no hot water is laid on, and there are no bathrooms. It seems that the expenditure required to provide boilers would have necessitated the reduction of the number of flats by ten. Leading out from the kitchen is a small coal cellar, and another door opens out on to a tiny balcony where a safe, suspended against the wall, makes up partially for the absence of a larder The kitchen is provided with a dust-shoot, a contrivance which has been condemned on sanitary grounds in some of the better-class flats. Gas and electric light are paid for by the occupiers. The electric light is supplied by means of a shilling in the slot arrangement. A nice garden, partly lawn, flower-bed and terrace is enclosed on three sides by the buildings. A bicycle shed and a fire-escape are among the useful accessories provided.

